# *EDR2 *negatively regulates salicylic acid-based defenses and cell death during powdery mildew infections of *Arabidopsis thaliana*

**DOI:** 10.1186/1471-2229-7-35

**Published:** 2007-07-06

**Authors:** Sonja Vorwerk, Celine Schiff, Marjorie Santamaria, Serry Koh, Marc Nishimura, John Vogel, Chris Somerville, Shauna Somerville

**Affiliations:** 1 Carnegie Institution, Department of Plant Biology, 260 Panama Street, Stanford CA 94305, USA; 2 Febit Biotech Gmbh, Heidelberg, Germany; 3 Alcimed, Paris, France; 4 Sogang University, Seoul, 100-611, South Korea; 5 Department of Biology, University of North Carolina, Chapel Hill, NC, USA; 6 USDA-ARS Western Regional Laboratory, Albany, CA, USA; 7 Department of Biological Sciences, Stanford University, Stanford CA 94305, USA

## Abstract

**Background:**

The hypersensitive necrosis response (HR) of resistant plants to avirulent pathogens is a form of programmed cell death in which the plant sacrifices a few cells under attack, restricting pathogen growth into adjacent healthy tissues. In spite of the importance of this defense response, relatively little is known about the plant components that execute the cell death program or about its regulation in response to pathogen attack.

**Results:**

We isolated the *edr2-6 *mutant, an allele of the previously described *edr2 *mutants. We found that *edr2-6 *exhibited an exaggerated chlorosis and necrosis response to attack by three pathogens, two powdery mildew and one downy mildew species, but not in response to abiotic stresses or attack by the bacterial leaf speck pathogen. The chlorosis and necrosis did not spread beyond inoculated sites suggesting that EDR2 limits the initiation of cell death rather than its spread. The pathogen-induced chlorosis and necrosis of *edr2-6 *was correlated with a stimulation of the salicylic acid defense pathway and was suppressed in mutants deficient in salicylic acid signaling. *EDR2 *encodes a novel protein with a pleckstrin homology and a StAR transfer (START) domain as well as a plant-specific domain of unknown function, DUF1336. The pleckstrin homology domain binds to phosphatidylinositol-4-phosphate *in vitro *and an EDR2:HA:GFP protein localizes to endoplasmic reticulum, plasma membrane and endosomes.

**Conclusion:**

*EDR2 *acts as a negative regulator of cell death, specifically the cell death elicited by pathogen attack and mediated by the salicylic acid defense pathway. Phosphatidylinositol-4-phosphate may have a role in limiting cell death via its effect on EDR2. This role in cell death may be indirect, by helping to target EDR2 to the appropriate membrane, or it may play a more direct role.

## Background

The hypersensitive necrosis response (HR) elicited by incompatible plant-pathogen interactions is thought to be a form of programmed cell death. Several of the features diagnostic for programmed cell death, such as nuclear condensation, DNA fragmentation and cytoplast shrinkage have been observed in plants cells undergoing HR [1].

Searches of sequenced plant genomes for plant orthologs of animal programmed cell death genes have identified only one gene that resembles its animal counterpart, the *BAX INHIBITOR 1 *gene, suggesting that components of the regulation and execution of programmed cell death differ substantially between animals and plants [2]. In spite of this conclusion, several observations suggest that plant and animal programmed cell death processes share some properties. Expression of the BAX pro-apoptotic factor in plants causes cell death and the plant BAX INHIBITOR 1 suppresses this cell death [3]. Inhibitors known to block the action of caspases in animals are effective at limiting HR in plants [4]. Recently, vacuolar processing enzyme gamma was identified as the functional equivalent of animal caspases [5,6]. In addition, *BECLIN1*, an ortholog of the yeast and animal autophagy genes *ATG6/BECLIN1*, was identified by the run-away cell death observed in *beclin1*-deficient mutants following pathogen attack. The ability of plant *BECLIN1 *to restrict cell death was dependent on several other autophagy-related genes providing another point of similarity between plant and animal programmed cell death [7]. Finally, sphingolipids have been implicated in cell death in both plants and animals. The fungal toxin fumonisin B1, which blocks ceramide biosynthesis in animals and elicits programmed cell death response, exerts a similar effect on plants [8]. Similarly, AAL toxin, a host-selective toxin produced by *Alternaria alternata *f. sp. *lycopersici *(a pathogen of tomato) causes cell death in both plants and animals and appears to target the same step in ceramide biosynthesis as fumonisin B1 [8,9]. In addition, the *acd5 *and *acd11 *mutants, which exhibit constitutive cell death, carry mutations in genes encoding a ceramide kinase and a sphingosine transfer protein, respectively [10,11].

These similarities are not sufficient to provide a complete understanding of programmed cell death or the HR in plants. Lesion mimic mutants, displaying spontaneous lesions, have been recovered in screens for mutants with deregulated cell death and have arisen in screens for mutants with altered disease resistance properties [1,12]. Among the cloned genes are those that resemble resistance genes (*SSI1, SSI4*) that appear to be constitutively activated. *COP *(copine, a Ca^+2 ^binding and phospholipid binding protein), *LSD1 *(Zn-finger domain, putative transcription factor), and barley *MLO *(a negative regulator of defenses against powdery mildews) may be involved in the signaling of cell death. Also, mutations in several metabolic genes (*DND1 *[cyclic nucleotide gated channel 2], *HLM1 *[cyclic nucleotide gated channel 4], *SSI2 (=FAB2*) [stearoyl-ACP desaturase]*, LIN2 *[coproporphyrinogen III oxidase], *ACD2 *[red chlorophyll catabolite reductase]) exhibit spontaneous lesions. Notable among these metabolic genes are the sphingolipid metabolism genes *ACD5 *and *ACD11 *mentioned above. In addition, mutations of genes encoding a number of novel proteins (ACD6 [ankyrin-repeat containing protein], SVN1 [GRAM domain containing membrane protein], and CPR5 [transmembrane protein]) lead to spontaneous lesioning phenotypes.

In addition to the lesion mimic mutants, a few mutants have been described that do not develop spontaneous lesions but rather display HR-like lesions only in response to a stimulus such as pathogen attack. *enhanced disease resistant 1 *(*edr1*)*-edr3 *are examples of such mutants [13-16]. *edr1 *and *edr2*, but not *edr3*, also show elevated defense responses (e.g., PR1 expression) following powdery mildew attack. These phenotypes were suppressed in mutants with defects in the salicylic acid (SA) signal transduction pathway (e.g., *pad4, npr1, eds1*) but not by those with defects in the ethylene/jasmonate pathway (i.e., *ein2*), suggesting that these mutants are hypersensitive to or have a lower threshold for responding to stress and activating the SA pathway. *EDR1 *encodes a CTR-like kinase, EDR2 a novel protein, and EDR3 a dynamin-like protein (DRP1E) [14,15,17]. The *edr1 *and *edr2 *mutants have a second phenotype that is SA-independent; they are hyper-sensitive to ethylene-induced senescence, implicating these two genes in the regulation of senescence as well as defense signaling [14,17]. The diverse nature of processes interrupted in these mutants suggests that much remains to be uncovered about the mechanisms controlling cell death in plants.

We initiated a screen for mutants that developed an exaggerated cell death response following inoculation with the powdery mildew fungus, *Golovinomyces cichoracearum *(=*Erysiphe cichoracearum*) as a means of identifying components of the HR programmed cell death. Lesion mimic mutants with spontaneous lesions were discarded from this screen to minimize the likelihood of recovering mutants with a metabolic dysfunction or that were compromised in the mechanisms protecting plants from the oxidative stress that arises during photosynthesis. These mutants were named *mildew-induced lesions *(*mil*) mutants and below we describe the characterization of the *mil1 *mutant and cloning of *MIL1 *gene. During the course of this work, *EDR2 *was cloned and as described below shown to be the gene compromised in the *mil1 *mutant [16]. For this reason, we have renamed *mil1 edr2-6*.

## Results

### *edr2 *exhibits a late acting resistance phenotype associated with cell death

**Figure 1:**
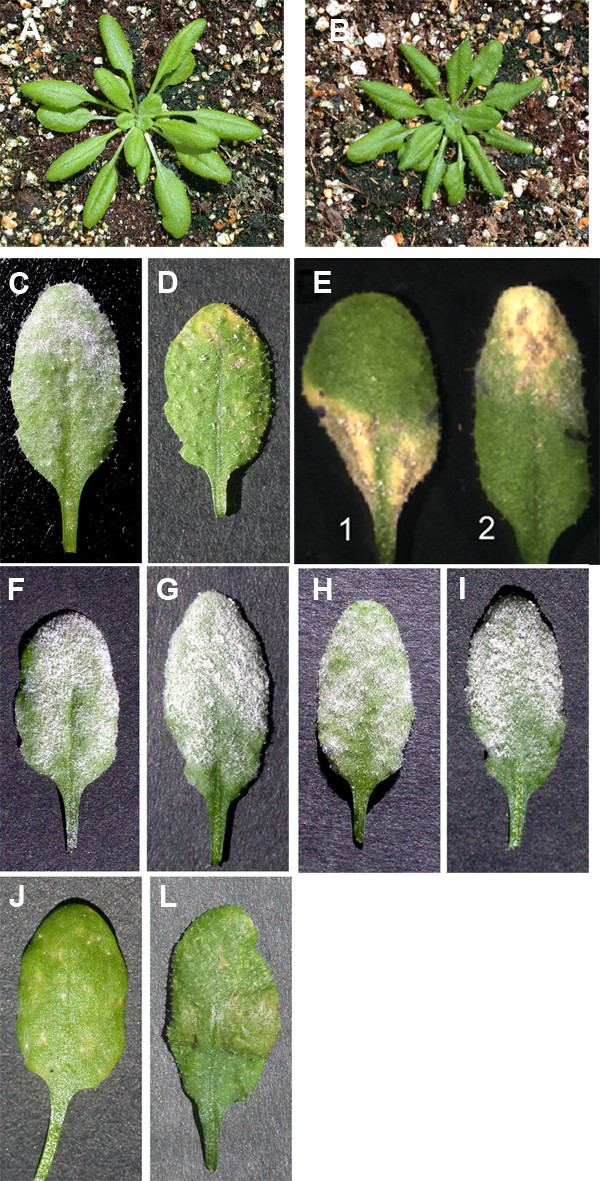
Macroscopic phenotypes of the *edr2-6 *mutant. (A, B) Uninfected plants at 25 d after germination; (A) wild type (B) *edr2-6*. (C, D, F-L) Three-week-old plants photographed at 7 dpi with *G. cichoracearum *(C) wild type, (D) *edr2-6*, (E) *edr2-6 *(1) The top-half or the (2) bottom-half of each leaf was covered with medical tape prior to inoculation. (F) *NahG*, (G) *pad4-1*, (H) *edr2-6 NahG*, (I) *edr2-6 pad4-1*, (J) *edr2-6 coi1-1*, (L) *edr2-6 ein2-1.*

The *edr2-6 *mutant was indistinguishable from wild type in growth and development up to ~3 weeks of age (Fig. 1A,1B). The wild type remained free of lesions whether inoculated with the powdery mildew pathogen or not (Fig. 1C). The mutant did not develop lesions spontaneously. It only became chlorotic and formed lesions at infection sites and did not support visible fungal growth (Fig. 1D,1E; see also Fig. 1a of Tang et al. [16]). The lesions on *edr2-6 *leaves did not spread to non-infected parts of inoculated leaves or to uninoculated leaves of the same plant (Fig. 1E). At later stages, the petioles of *edr2-6 *leaves were slightly shorter and the leaves tended to be crinkled (Fig. 1A,1B).

The severity of the *edr2-6 *phenotype varied with inoculation density. Fungal growth measurements up to 5 days post-inoculation (dpi) obtained under very low inoculation densities (~1 conidium per mm^2^) are similar on Col-0 and *edr2-6 *(data not shown). Similarly, their macroscopic phenotypes were identical up to this time point (data not shown). By contrast, when fungal growth was monitored at 7 dpi under high inoculum density (~100 conidia per mm^2^), the mutant and wild type were clearly distinguished with wild type supporting significantly more fungal growth than *edr2-6*. Under these conditions, wild-type leaves had an average of 236 ± 87 conidiophores per mm^2^, whereas the *edr2-6 *mutant had 49 ± 40 conidiophores per mm^2 ^(average ± standard deviation, n = 75, p ≤ 0.01 by Student's t-test).

The timing of macroscopic lesion formation in the mutant varied with inoculation density and, as also reported by Tang et al. [16], occurred relatively late in the infection cycle compared to the rapid HR (<24 hours post-inoculation [hpi]) typically elicited by incompatible interactions governed by plant resistance and pathogen avirulence genes. Macroscopically, the first lesions appeared 4 dpi at high inoculation densities and 7 dpi at low inoculation densities. The wild-type plants did not develop visible lesions upon powdery mildew infection regardless of inoculum density. Fungal infection eventually led to an apparent acceleration of senescence in a density-dependent fashion in both wild type and mutant, but the amount of chlorosis was greater in the mutant. At 7 dpi, leaves infected with ~100 conidia per mm^2^, had 6.0% ± 6.2% chlorotic tissue in the wild type, whereas the *edr2-6 *leaves had 22.1% ± 13.9% chlorotic and 10.6% ± 5.6% necrotic tissue (average ± standard deviation, n = 15 leaves). The amount of necrotic tissue in the mutant also correlated with the inoculation density. Thus, at 7 dpi with ~20 conidia per mm^2^, the *edr2-6 *mutant had 14.3% ± 10.7% chlorotic tissue and 2.6% ± 2.3% necrotic tissue (average ± standard deviation, n = 10 leaves).

**Figure 2:**
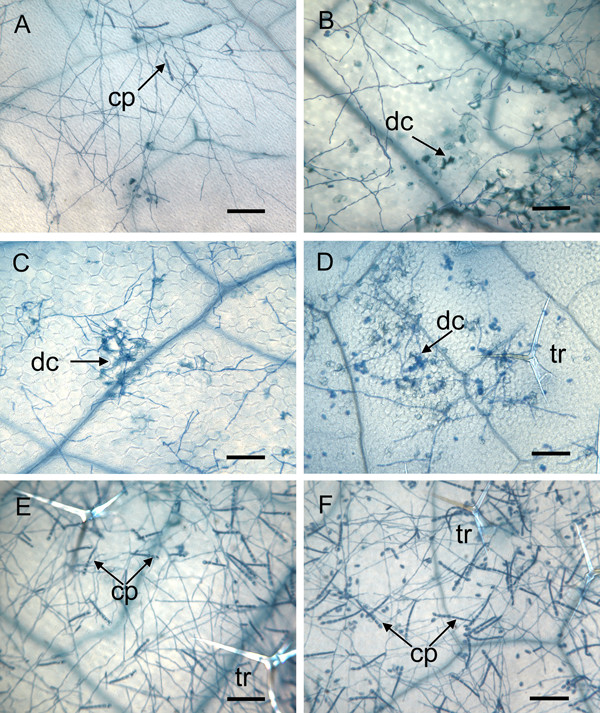
Microscopic visualization of fungal growth and cell death on leaves of 3-week-old plants at 7 dpi with *G. cichoracearum*. Leaves were stained with trypan blue. (A) wild type, (B) *edr2-6*, (C) *edr2-6 coi1-1*, (D) *edr2-6 ein2-1*, (E) *edr2-6 NahG*, (F) *edr2-6 pad4-1*. cp, conidiophores bearing asexual conidia; dc, dead cells; tr, trichome. Bar = 22 μm (A, B, D, F), 40 μm (C) and 26 μm (E).

Cell death was also monitored microscopically at various time points during the infection process under high inoculation density. In wild-type plants, no macroscopic lesions were observed upon inoculation with *G. cichoracearum *and only rare groups of more than three collapsed mesophyll cells were observed 7 dpi (Fig. 2A). Up to 2 dpi, *edr2-6 *leaves were indistinguishable from wild type, with no apparent cell death. By 3 dpi, a few isolated epidermal and mesophyll cells appeared dead in *edr2-6 *and were usually associated with fungal hyphae. The first small groups of dead mesophyll cells (2 to 3) also appeared 3 dpi. From 4 to 7 dpi, these lesions were more frequent, and increased in size (up to 50 to 100 cells) (Fig. 2B; see also Fig. 1D of Tang et al. [16]).

**Figure 3:**
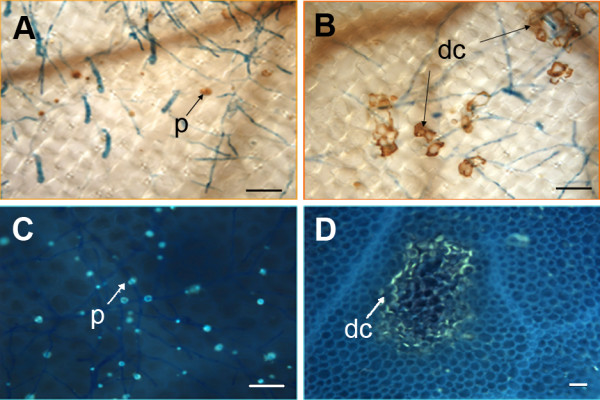
Hydrogen peroxide and callose accumulation in *edr2-6*. Three-week-old plants were inoculated with *G. cichoracearum *and stained for either hydrogen peroxide (A, B) or callose (C, D). (A, C) Col-0, (B, D) *edr2-6*. In D, callose outlines dead mesophyll cells in *edr2-6*. dc, dead cells; p, papilla. Bar = 22 μm.

The occurrences of hydrogen peroxide and callose, which typically accumulate in cells that undergo an HR, were assessed. In both wild-type and *edr2-6 *plants, hydrogen peroxide and callose were present at the fungal penetration sites (Fig. 3). In wild type, both compounds were found in papillae, cell wall appositions deposited by the plant at sites of attempted penetration. Both compounds also accumulated in whole cells, predominantly in *edr2-6 *leaves, following the pattern already observed for the appearance of dead cells. Autofluorescent compounds, believed to be antimicrobial molecules, also accumulated in whole *edr2-6 *cells in a similar pattern to that observed for the appearance of dead cells (data not shown).

### Lesion formation in *edr2-6 *is only induced by biotic stresses

**Figure 4:**
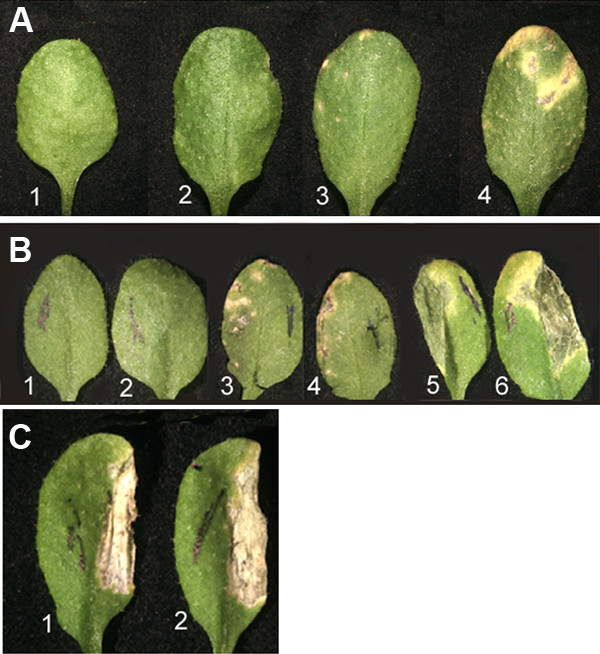
Response of *edr2-6 *to pathogens. (A) Leaves from plants 7 dpi with the barley pathogen, *Blumeria graminis *f.sp. *hordei*. (1) Col-0, inoculation density ~50 conidia per mm^2^, (2) *edr2-6*, inoculation density ~1 conidia per mm^2^, (3) *edr2-6*, inoculation density ~15 conidia per mm^2^, (4) *edr2-6*, inoculation density ~50 conidia per mm^2^. (B) Leaves at 2 dpi with *P. syringae *tomato DC3000. (1, 3, 5) Col-0, (2, 4, 6) *edr2-6*. Inoculation titers: (1,2) 10^2 ^cfu per ml; (3,4,) 10^4 ^cfu per ml; (5,6) 10^8 ^cfu per ml. (C) Leaves at 2 dpi with 10^8 ^cfu of *P. syringae *tomato DC3000 (*avrRpt2*). (1) Col-0, (2) *edr2-6*. Plants were 3-weeks old at the time of inoculation.

*Blumeria graminis *f.sp. *hordei*, the barley powdery mildew, which is not a pathogen of Arabidopsis, was also able to induce macroscopic lesion formation in *edr2-6*, but not Col-0 (Fig. 4A). In *edr2-6 *mutants, the number of dead cells was comparable to wild type until 3 dpi. By 4 dpi, the first small lesions occurred at high inoculation densities and increased in size until 7 dpi (Fig. 4A).

To ascertain whether lesion formation on *edr2-6 *leaves was specifically triggered by pathogen infection, plants were treated with several types of abiotic stress (mechanical, thermal, drought and light stress). No macroscopic or microscopic lesions were observed after any of these treatments as determined by visual observation and trypan blue staining (data not shown). After wounding, the amount and the localization of dead cells were comparable in Col-0 and in *edr2-6*, and no spreading cell death was observed in the mutant.

### *edr2-6 *mutants are resistant to some but not all pathogens

A number of lesion mimic mutants exhibit resistance to a broad spectrum of pathogens. To determine the resistance specificity of *edr2-6*, mutant plants were challenged with an oomycete pathogen, *Hyaloperonospora parasitica*, and a bacterial pathogen, *Pseudomonas syringae *pv tomato DC3000. The level of *edr2-6 *resistance to a biotrophic *H. parasitica *Emco5 was evaluated by counting the number of sporangia per cotyledon at 9 dpi. At high inoculum concentration (3 × 10^5 ^sporangia per ml), the wild type had 8.4 ± 4.9 sporangia per cotyledon, whereas the *edr2-6 *mutant had 3.5 ± 2.3 (average ± standard deviation, n = 30, p ≤ 0.01 by Student's t-test). At lower inoculum concentrations (10^5 ^sporangia per ml), wild type and mutant were indistinguishable (2.5 ± 2.4 and 2.0 ± 1.8 sporangia per cotyledon, respectively [average ± standard deviation, n = 30], p = 0.40 by Student's t-test). A second *H. parasitica *strain, Noco2, showed reduced growth and elicited lesions when inoculated onto the leaves of 3-week-old *edr2-6 *plants. The number of spores per mm^2 ^were 12.8 ± 10.5 (n = 15) and 6.0 ± 4.5 (n = 18) for wild type and *edr2-6*, respectively (p = 0.03 by Student's t-test).

*P. syringae *tomato DC3000 multiplies in both Col-0 and *edr2-6 *plants, eventually producing water-soaked lesions. Unlike the response to powdery mildew, the timing and extent of macroscopic and microscopic symptom development were similar for mutant and wild-type plants, regardless of the concentration of inoculum (Fig. 4B). The estimation of bacterial titers in the infected leaves did not reveal any significant difference between bacterial multiplication rates in Col-0 and *edr2-6 *(growth curves not shown). The *edr2-6 *plants, which carry the *RPS2 *resistance gene, mounted a normal hypersensitive necrosis response following infiltration with the avirulent bacterial strain carrying the *AvrRpt2 *gene (Fig. 4C). Similar results were reported by Tang et al. [16]. The loss of EDR2 function did not interfere with the ability of the plants to mount a normal HR.

### *edr2-6 *plants do not exhibit constitutively active defense responses

**Figure 5:**
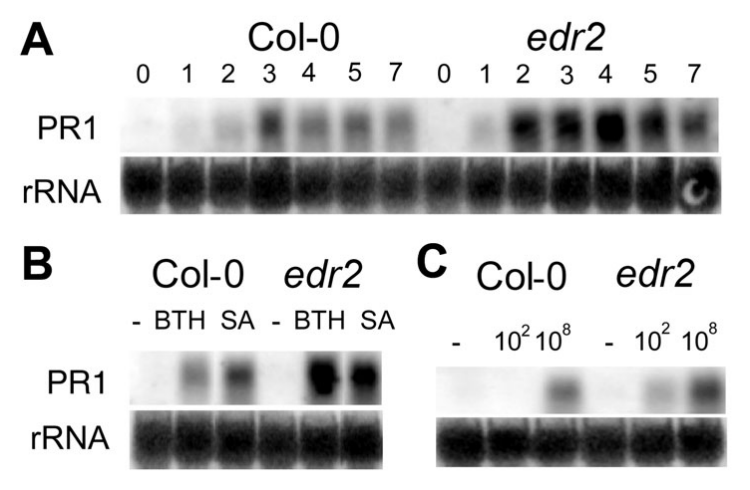
The expression of PR1 is enhanced in *edr2-6*. (A) Northern blot showing PR1 expression at various times (in days) following inoculation of Col-0 or *edr2-6 *with *G. cichoracearum*. (B) PR1 expression in Col-0 and *edr2-6 *at 2 days following treatment with water (-), 0.3 mM BTH or 0.5 mM SA. (C) PR1 expression in Col-0 and *edr2-6 *at 48 hpi with 0, 10^2 ^or 10^8 ^cfu per ml of *P. syringae *pv tomato DC3000.

Some lesion mimic mutants are disease resistant because defenses, including the SA signal transduction pathway, are constitutively activated [1,12]. Similar to the results of Tang et al. [16], *PR1 *transcript levels, a marker for the SA pathway, in uninfected *edr2-6 *plants were negligible and similar to uninfected wild-type plants, indicating that the SA pathway was not constitutively activated in the mutant. After 2 dpi, *PR1 *expression was induced in both wild-type and mutant plants and *PR1 *levels remained high up to 7 dpi (Fig. 5A). The level of *PR1 *induction was two-fold higher in *edr2-6 *relative to Col-0 plants at every time point suggesting possibly that *edr2-6 *mutants are predisposed to respond to a stimulus activating the SA pathway. This stimulus may be lesion formation as SA levels increase following treatments that induce lesions [12].

*PR1 *levels were also monitored in plants treated with SA and the SA mimic, BTH. As expected, these treatments induced *PR1 *expression in both Col-0 and *edr2-6*. However, in the mutant plants, *PR1 *gene expression was two-fold higher than in wild-type plants (Fig. 5B). The same trend in *PR1 *up-regulation occurred in *edr2-6 *plants infected with the virulent bacterium *P. syringae *tomato DC3000, in a dosage dependent manner (Fig. 5C).

The transcript levels of the *PDF1.2 *gene encoding an anti-microbial defensin, a marker for the jasmonate/ethylene signal transduction pathway, over the time course used for *PR1 *gene expression analysis were not significantly different between *edr2-6 *and Col-0 (data not shown).

### Both the resistance and lesion phenotypes are dependent on the SA pathway

To analyze the involvement of the major defense signaling pathways, double mutants with defects in the SA or jasmonate/ethylene pathways were analyzed for their resistance and lesion phenotype. Resistance was lost in plants expressing the *NahG *gene and in the *edr2-6 pad4-1 *double mutant (Fig. 1F,1G,1H,1I; see also Fig. 3 of Tang et al. [16]). Similar to *edr2-6*, the double mutants *edr2-6 coi1-1 *and *edr2-6 ein2-1 *did not support fungal growth and showed lesion formation (Fig. 1J,1L; see also Fig. 3 of Tang et al. [16]). At the microscopic level, the lesion phenotype was not suppressed by the *coi1-1 *or *ein2-1 *mutations (Fig. 2C,2D), but was lost in *edr2-6 *plants expressing *NahG *and in the *edr2-6 pad4-1 *double mutant (Fig. 2E,2F). Thus, the SA pathway contributes to lesion formation and the resistance phenotype in *edr2-6 *mutants. Since *G. cichoracearum *is an obligate biotrophic pathogen, requiring living host tissue for growth, the resistance phenotype is likely a consequence in part of the inability of the chlorotic and lesioned tissue to support growth of this pathogen.

### *EDR2 *encodes a novel, ubiquitously expressed protein

The segregation analysis of F_2 _plants from a cross between *edr2-6 *and wild type suggested that the *edr2-6 *mutation was linked to a single T-DNA insertion, and that both the disease resistance and the lesion phenotypes of *edr2-6 *following powdery mildew infection were due to a unique recessive mutation (data not shown). The *EDR2 *gene was isolated by cloning the regions flanking the T-DNA insert. Sequencing revealed that the T-DNA was inserted in a predicted intron of gene At4g19040, a gene cloned previously as *EDR2 *[16]. A 12 kb fragment covering this putative gene was cloned into a binary Ti plasmid and used to transform homozygous *edr2-6 *plants. The wild-type phenotypes (susceptibility and no lesions following powdery mildew inoculation) were restored in 54 (98.2%) of the 55 T_1 _plants tested (data not shown). The progeny of six of these T_1 _plants segregated 3:1 (susceptible:resistant) for powdery mildew resistance, as expected.

**Figure 6:**
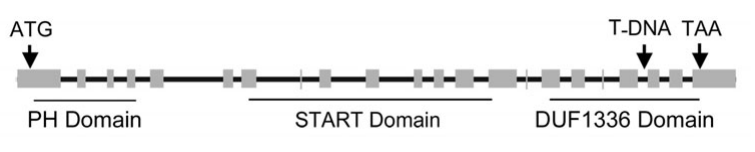
Predicted *EDR2 *gene and protein structure. Intron-exon structure of the *EDR2 *gene. The regions of the gene corresponding to the PH, START and DUF1336 domains are indicated by lines and the site of the T-DNA insertion in the *edr2-6 *mutation is indicated by an arrow along with the ATG start codon and TAA stop codon.

A cDNA for the *EDR2 *gene was isolated by RT-PCR. Its sequence was identical to the NCBI deduced cDNA sequence NM_118022. The genomic sequence of *EDR2*, which is composed of 22 exons and 21 introns, extends 5,373 nucleotides. The coding sequence is 2,157 nucleotides long and encodes for a protein of 718 amino acids with a predicted molecular weight of 82 kD. The EDR2 protein consists of an N-terminal pleckstrin homology (PH) domain (2.6 × e^-9 ^confidence value), a central region with a StAR-related lipid-transfer (START) domain (1.8 × e^-8^) and a C-terminal, plant-specific, domain of unknown function, DUF1336 (1.5 × e^-115^) (Fig. 6) [18].

A gene on chromosome V, At5g45560, is homologous to *EDR2 *with >75% nucleotide identity across the entire gene and approximately 89% identity on the protein level. Two other genes in the Arabidopsis genome, At2g28320 and At3g54800, are predicted to encode proteins that display the same domain structure as EDR2 with PH, START and DUF1336 domains. These proteins show relatively little sequence similarity to EDR2 (36% and 32% amino acid sequence identity, respectively).

A survey of published gene expression profiling data showed that *EDR2 *is expressed in all organs [19], corroborating pEDR2:GUS expression results from Tang et al. [16]. Its expression did not vary substantially during development, with the exception that in stamens and senescing leaves, *EDR2 *transcript levels were ~2–3-fold higher than in most other organs or developmental stages (Table 1). As observed by Tang et al. [16], *EDR2 *transcript levels were generally unresponsive to biotic stresses. The highest inductions (~2.2-2.3 fold) were elicited by inoculation with the necrotrophic fungal pathogen, *Botrytis cinerea*, at 48 hpi and by the bacterial pathogen, *P. syringae *tomato DC3000, at 24 hpi (Table 2). In contrast, At5g45560 transcript levels were generally ~1/3 those of *EDR2 *during development and in various organs, and mostly below the level of reliable detection in the biotic stress experiments. Transcript levels of At3g54800 were very low and not detected in most organs, developmental stages or under various biotic stresses, with the exception of the stamens, which expressed this gene at levels ~100-fold higher than in most other organs. At2g28320 was expressed at ~1/2 the level of *EDR2 *with its highest transcript levels occurring in mature flower parts. The expression of this latter gene was unresponsive to biotic stresses.

**Table 1:** Expression values for *EDR2 *(At4g19040) and related genes in different plant organs at varying developmental stages

**Stage **^a^	**At4g19040**	**At2g28320**	**At3g54800**	**At5g45560**
	254602_at ^b^	265273_at	251854_at	248948_at
	Signal Value ^c^			
*Experiment 87*				
ATGE_2_A_hypocotyl	1,699	1,198	65	513
ATGE_3_A_roots	1,601	719	66	746
ATGE_4_A_shoot_apex	1,323	680	ns ^d^	333
ATGE_5_A_leaves_1+2	1,310	672	ns	ns
ATGE_6_A_shoot_apex	1,361	710	83	522
ATGE_7_A2_seedling_green	1,613	1,076	ns	311
*Experiment 88*				
ATGE_8_A_shoot_apex	1,856	835	ns	847
ATGE_9_A_roots	1,286	699	81	710
ATGE_10_A_rosette_leaf	1,213	606	ns	327
ATGE_12_A_rosette_leaf_2	1,847	891	93	ns
ATGE_13_A_rosette_leaf_4	1,339	850	ns	216
ATGE_14_A_rosette_leaf_6	1,291	896	ns	ns
ATGE_15_A_rosette_leaf_8	1,144	740	91	262
ATGE_16_A_rosette_leaf_10	1,135	681	ns	ns
ATGE_17_A_rosette_leaf_12	1,189	640	ns	274
ATGE_19_A_leaf7_petiol	1,244	641	ns	341
ATGE_20_A_leaf7_prox_half	1,234	743	ns	ns
ATGE_21_A_leaf7_dist_half	1,212	868	ns	ns
ATGE_22_A_whole_plant	1,413	696	ns	303
ATGE_23_A_whole_plant	1,445	712	ns	318
ATGE_24_A_whole_plant	1,580	807	ns	343
ATGE_25_A_senescing_leaf	3,573	1,843	144	ns
ATGE_26_A_cauline_leaf	2,058	1,109	ns	ns
ATGE_27_A_stem	2,558	1,476	ns	465
ATGE_28_A2_1st_node	1,858	1,269	ns	661
ATGE_29_A2_shoot_apex	1,308	852	65	851
*Experiment 89*				
ATGE_31_A2_flower_stage_9	1,377	968	ns	741
ATGE_32_A2_flower_stage_10/11	1,534	1,109	87	548
ATGE_33_A_flower_stage_12	1,975	1,143	908	623
ATGE_34_A_stage_12_sepal	2,234	1,063	257	ns
ATGE_35_A_stage_12_petal	1,941	1,576	429	621
ATGE_36_A_stage_12_stamen	3,557	1,893	8,983	1,180
ATGE_37_A_stage_12_carpel	1,773	875	125	858
ATGE_39_A_flower_stage_15	2,581	1,477	3,605	238
ATGE_40_A_stage_15_pedicel	1,502	662	171	396
ATGE_41_A_stage_15_sepal	3,192	2,279	704	ns
ATGE_42_B_stage_15_petal	3,440	2,782	764	ns
ATGE_43_A_stage_15_stamen	4,898	2,248	15,399	ns
ATGE_45_A_stage_15_carpel	1,738	756	797	609

Median	1,580	868	157	513

^a ^From Genevestigator [19], AtGenExpress Experiments Developmental Baseline I (87), II (88) and II (89) from Schmid et al. [62]. ^b ^Affymetrix probe set identifier. ^c ^Average of 3 replicates. ^d ^ns, no signal. Less than 3 replicates with well measured values (i.e., p-value </= 0.06) available.

**Table 2:** Fold-change in the expression of EDR2 (At4g19040) and related genes following inoculation of wild-type plants with selected pathogens

**Treatment **^a^		**At4g19040**	**At2g28320**	**At3g54800**	**At5g45560**
*Numerator*	*Denominator*	251854_at^b^	265273_at	254602_at	248948_at

Exp. 146, Time course of infection with *Golovinomyces orontii*		Ratio ^c^			
ATGE_EOr_6 h_inf	ATGE_EOr_6 h_uninf	1.1	1.0	ns ^d^	ns
ATGE_EOr_12 h_inf	ATGE_EOr_12 h_uninf	0.9	0.9	ns	ns
ATGE_EOr_18 h_inf	ATGE_EOr_18 h_uninf	1.0	0.8	ns	ns
ATGE_EOr_24 h_inf	ATGE_EOr_24 h_uninf	1.0	0.9	ns	ns
ATGE_EOr_48 h_inf	ATGE_EOr_48 h_uninf	1.0	1.0	ns	ns
ATGE_EOr_72 h_inf	ATGE_EOr_72 h_uninf	0.6 ^e^	0.8	ns	ns
ATGE_EOr_96 h_inf	ATGE_EOr_96 h_uninf	0.9	0.9	ns	ns
ATGE_EOr_120 h_inf	ATGE_EOr_120 h_uninf	0.7	0.8	ns	ns
					
Exp. 147, *Botrytis cinerea *infection					
ATGE_Bcin_inf_48 h	ATGE_Bcin_con_48 h	2.2 ^e^	1.7	ns	ns
					
Exp. 106, *Pseudomonas syringae *infections					
ATGE_Psyr_phaseol_24 h	ATGE_Psyr_MgCl2_24 h	1.3	ns	ns	ns
ATGE_Psyr_HrcC-_24 h	ATGE_Psyr_MgCl2_24 h	1.0	ns	ns	ns
ATGE_Psyr_DC3000_24 h	ATGE_Psyr_MgCl2_24 h	2.3 ^e^	ns	ns	ns
ATGE_Psyr_avrRpm1_24 h	ATGE_Psyr_MgCl2_24 h	1.4	ns	ns	ns

a Data recovered from Genevestigator [19]. The data from experiment 106 are from the Nürnberger laboratory and experiments 146 and 147 are from the Ausubel laboratory. b Affymetrix probe set identifier for the ATH1 GeneChip. c Ratio of the average signal value for the given numerator treatment over the average signal value for the given denominator treatment. d ns, no signal. Less than 3 replicates with well measured values (i.e., p-value </= 0.06) available for the numerator, the denominator or both. e Expression levels from inoculated and uninoculated plants were significantly different (Student's t-test, p = 0.01).

### The PH domain of EDR2 specifically binds to phosphatidylinositol-4-phosphate *in vitro*

**Figure 7:**
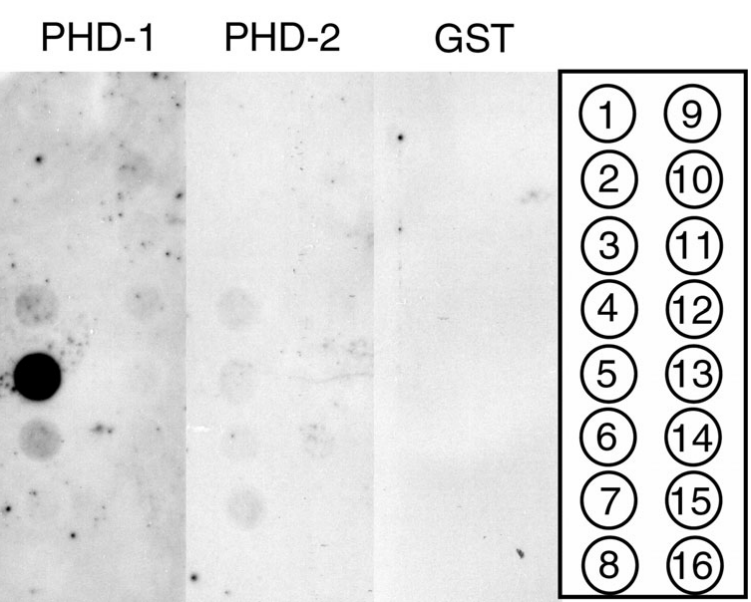
Binding of the EDR2 PH-domain to lipids. GST-tagged EDR2 PH domain was affinity purified and used to probe blots spotted with various lipids. The PH-GST fusion proteins were detected with an anti-GST antibody. PHD-1: wild-type PH-domain of EDR2; PHD-2: mutated PH-domain of EDR2 (F93S); GST: glutathione S-transferase negative control. Compounds spotted on the membrane: 1, lysophosphatidic acid; 2, lysophosphatidylcholine; 3, phosphatidyl inositol; 4, phosphatidylinositol 3-phosphate; 5, phosphatidylinositol 4-phosphate; 6, phosphatidylinositol-5-phosphate; 7, phosphatidyl ethanolamine; 8, phosphatidyl choline; 9, sphingosine-1-phosphate; 10, phosphadidylinositol-3,4-phosphate; 11, phosphadidylinositol-3,5-phosphate; 12, phosphatidylinositol-4,5-phosphate; 13, phosphadidylinositol-3,4,5-phosphate; 14, phosphatidic acid; 15, phosphatidyl serine; 16, blank

The PH domain of EDR2, expressed as a PH domain-GST fusion protein, had strong *in vitro *binding affinity for phosphatidylinositol-4-phosphate (PI-4-P) (Fig. 7). Very weak binding to phosphatidylinositol-3-phosphate or phosphatidylinositol-5-phosphate was also observed. In the course of cloning the PH domain, we fortuitously obtained a mutant PH-GST construct in which the phenylalanine in position 93 was replaced by a serine. This fusion protein completely lacked the ability to bind to PI-4-P, suggesting that this amino acid is important for the PI-4-P binding (Fig. 7).

### EDR2 is localized to multiple compartments

**Figure 8:**
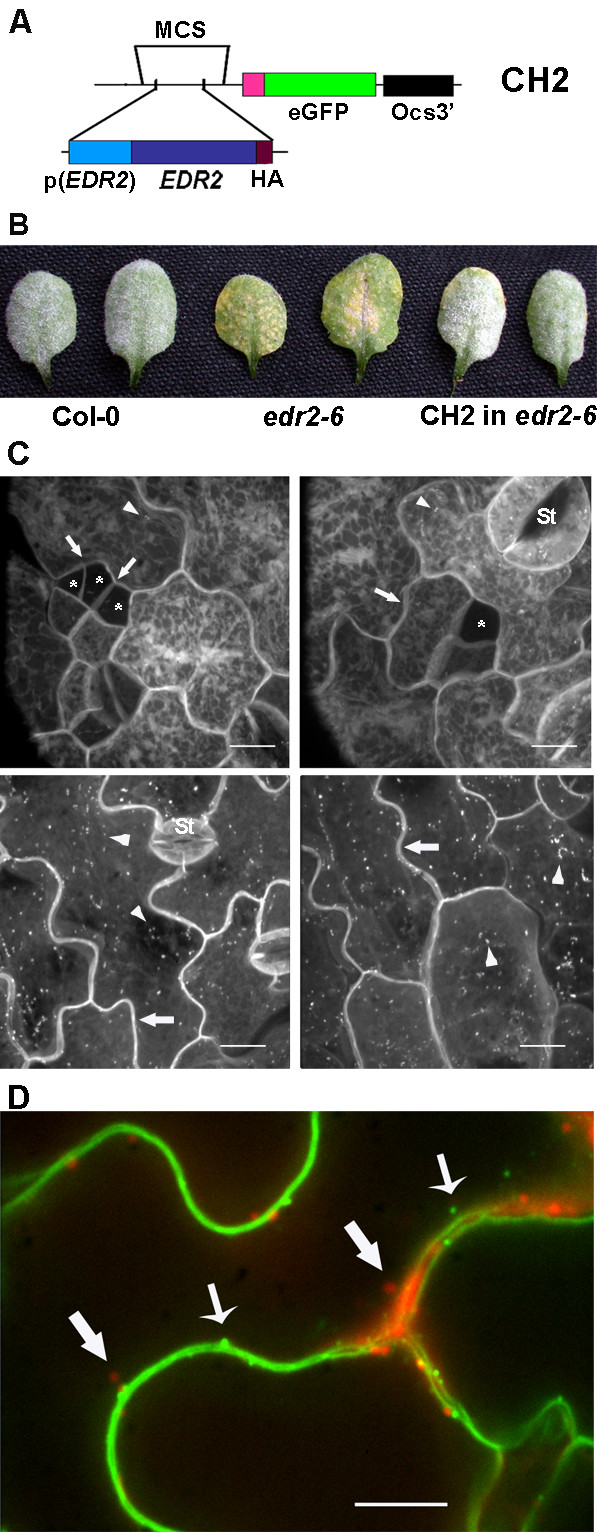
EDR2 localizes to multiple subcellular compartments. (A) EDR2:HA:eGFP construct, CH2. Note, the eGFP sequence included the sequence for 10 Ala at the N-terminus (pink block). (B) EDR2:HA:GFP (labeled CH2) restores the *edr2-6 *mutant to disease susceptibility and suppresses the chlorosis and necrosis phenotype. For each genotype, two leaves from 3-week-old plants are shown at 7 dpi with *G. cichoracearum*. (C) EDR2:HA:eGFP was localized mainly to the endoplasmic reticulum as shown by the fluorescently-labeled reticulate net-like structure. EDR2:HA:eGFP also localized to the plasma membrane (arrows) and to endosomes (arrowheads). The upper two panels are from cotyledons of 7-day-old seedlings. Young, recently divided cells (asterisk) exhibited reduced EDR2:HA:eGFP fluorescence compared to more mature cells, including stomatal guard cells. The lower two panels are from leaves from 7-week old plants. St, stomata. Bars = 13.4 μm. (D) EDR2:HA:eGFP and the MitoTracker dye stain different sub-cellular structures. Merged image of the MitoTracker image (red) and the EDR2:HA:eGFP image (green). Thick arrows point to small bodies, mitochondria, stained with the MitoTracker dye and thin arrows point to endosomes tagged with EDR2:HA:eGFP. EDR2:HA:eGFP also localizes to the plasma membrane. Bars = 13.4 μm.

A C-terminal eGFP fusion construct with expression driven by the native *EDR2 *promoter was transformed into *edr2-6 *and the resulting lines were analyzed for complementation of the mutant phenotype and GFP fluorescence (Fig. 8A). The construct complemented both the resistance and the lesion phenotype in five independent transgenic lines (Fig. 8B). Using a spinning disk scanning laser confocal microscope, EDR2:HA:eGFP was observed in the endoplasmic reticulum, plasma membrane and in small endosomes in young seedlings (Fig. 8C, upper panels). In young dividing cells, the expression of EDR2 seemed greatly reduced relative to levels observed in more mature cells (Fig. 8C, asterisked cells). In the rosette leaves of mature plants, EDR2:HA:eGFP was localized to the same three subcellular compartments, although to a lesser relative extent to the endoplasmic reticulum. EDR2:HA:eGFP did not co-localize with the mitochondrial dye MitoTracker (Fig. 8D).

## Discussion

The *edr2 *mutants exhibit properties consistent with the assumption that EDR2 acts as a negative regulator of cell death ([16], this publication). The chlorosis and necrosis phenotypes do not develop spontaneously and do not develop in response to various abiotic stresses, such as wounding, heat stress, light stress, or drought stress. The chlorosis and necrosis were elicited only following inoculation with the pathogens *G. cichoracearum*, *B. g. hordei *or *H. parasitica *(Fig. 1D,1E, 2B; and [16]). These results confirm that *EDR2 *plays a role specifically in the cell death associated with plant-pathogen interactions and does not have a general role in cell death. These features distinguish the *edr2 *mutants from typical lesion mimic mutants such as the *acd *and *lsd *classes. In addition, the occurrence of chlorotic and necrotic tissue was restricted to inoculation sites and did not spread suggesting that EDR2 restricts the initiation of cell death rather than its spread (Fig. 1E).

Because both *G. cichoracearum *and *H. parasitica *are biotrophic pathogens, the chlorosis and necrosis that develop may be sufficient to account for the restricted growth of these pathogens in the *edr2 *mutants. However, the SA pathway, but not the ethylene/jasmonate pathway, appears to be somewhat deregulated in that PR1 transcript levels are elevated in *edr2 *mutants relative to wild type following elicitation by BTH or pathogen attack (Fig. 5; [16]). Furthermore, plants deficient in SA accumulation or signaling suppress both the development of chlorosis and necrosis as well as the disease resistance phenotypes of *edr2 *mutants (Fig. 1H,1I, 2E,2F; and [16]). Thus, it is also possible that SA-dependent defenses unrelated to cell death contribute to the disease resistance phenotype of *edr2 *mutants.

The SA signal transduction pathway is required for the HR elicited by incompatible plant-pathogen interactions. However, cell death is known to activate the SA signal transduction pathway in adjacent living tissue in a positive feedback loop that amplifies signal transduction via this defense pathway [12]. Thus, it is difficult to know whether EDR2 acts upstream of SA to limit SA activation of cell death or downstream of SA. PAD4 and EDS1 have homology to lipases and have been shown to be required for the accumulation of SA [20,21]. Given that EDR2 may bind lipid-like molecules via both its PH and START domains, EDR2 may have a direct or indirect inhibitory effect on PAD4 or EDS1 via a lipid-like intermediate. Candidates for this lipid-like intermediate could be a sphingolipid [10,11], phosphatidic acid [22-26] or oleic acid [27].

EDR2 encodes a novel protein with three predicted domains, a PH, a START and a DUF1336 domain. Three other predicted proteins with this domain structure occur in the Arabidopsis genome and three in the rice genome (XP_463792, NP_922009, ABB47745), but none have been assigned a function to date [28]. The DUF1336 domain appears to be plant-specific but 27 animal proteins contain PH and START domains including the human CERT (AAR26717), a splice variant of the Goodpasture antigen binding protein [28]. In the CERT protein, the PH domain binds PI-4-P as does the EDR2 PH domain [29]. In addition, the START domain of the CERT protein binds to ceramides. From these properties, Hanada et al. (2003) suggested that this protein acts to carry ceramides via a non-vesicle mediated transport mechanism from their site of synthesis in the endoplasmic reticulum to the Golgi where they are converted to syphingomyelin [29].

Plants also synthesize ceramide and more complex sphingolipids, some of which have been localized to detergent-resistant membrane domains [30-32]. Alterations in ceramides and/or sphingolipids or possibly the accumulation of their precursors stimulate cell death in plants and animals. The mechanism by which sphingolipids promote cell death is unknown and may be indirect via their impact on the functioning of cell death effectors found in lipid rafts such as ion channels [33]. It is tempting then to speculate that EDR2, like CERT, carries ceramides from the endoplasmic reticulum to another subcellular membrane, such as the plasma membrane or endosomes. Both membranes are labeled by EDR2:HA:GFP (Fig. 8). Presumably, the vesicle-mediated movement of ceramides among plant compartments is sufficient to support normal growth and development. If responses to pathogen attack demand additional ceramides or sphingolipids in a specific membrane, then non-vesicle-mediated transport via EDR2 may be required to supplement vesicle-mediated transport. This might explain why *edr2 *mutants do not constitutively exhibit lesions, as do *acd5 *and *acd11*. It is also possible that At5g45560, the closely related gene, is partially redundant to *EDR2 *but unable to meet extra demand in plants under pathogen attack.

Alternate models are possible. The function(s) of PH domains is not clear, but it is generally believed that they provide a way to selectively direct proteins to membranes [25]. However, the PH-domain can bind ligands other than phosphatidylinositols. For example, the PH-domains of the β-adrenergic receptor kinase and phospholipase C β bind to both a lipid and the G_β,γ _subunit of trimeric G-proteins [34,35]. Furthermore, it is conceivable that the PH-domain may interact co-operatively with the START-domain and that the concerted action of both domains influences the ligands bound to EDR2 and consequently EDR2 function, as has been observed with the insulin receptor substrate 1 [36]. It is equally possible that the DUF1336 domain plays a novel role in restricting cell death [16] and the PH and START domains serve to localize the EDR2 protein to the correct membrane following pathogen attack or during senescence. Determining the lipid or sterol molecule bound by the START domain would be an important step in unraveling the role of EDR2 in restricting cell death in plant-pathogen interactions.

## Conclusion

*EDR2 *was isolated as a negative regulator of cell death, specifically the cell death elicited by pathogen attack but not by abiotic stresses. EDR2 encodes a novel protein with PH, START and DUF1336 domains. The PH domain of EDR2 binds preferentially to PI-4-P and the EDR2 protein localizes to endosomes, the endoplasmic reticulum and the plasma membrane. The lesions that develop in *edr2 *mutants are dependent on the SA signal transduction pathway providing an additional link to defense responses. Thus, it is possible that EDR2 or possibly a lipid/sterol product acts in opposition to the SA pathway to fine tune the HR.

## Methods

### Biological materials and growth conditions

The *edr2-6 *mutant is a T-DNA insertion mutant derived from Col-0 [37] and was backcrossed to Col-0 once. The plants were grown in growth chambers at 22°C with a 14-h photoperiod, except for those plants to be inoculated with *Hyaloperonospora parasitica*, which were grown at 16°C in a 10-h photoperiod. The maintenance of the *G. cichoracearum *UCSC1, the production of inoculum on a secondary host, squash (variety Kuta), and the inoculation procedures were previously described [38,39]. The barley powdery mildew, *Blumeria graminis *f.sp. *hordei *race CR3, was maintained on barley variety CI-16138 (=AlgerianS) and inoculated onto barley or Arabidopsis plants as described [40]. Maintenance and infiltration with *Pseudomonas syringae *pv tomato DC3000 [41] and inoculation with *H. parasitica *Emco5 [42] were performed as described by Vogel and Somerville [37]. Bacterial growth curves were performed by estimating the titers of bacteria in leaves up to 4 dpi [13].

Some powdery mildew inoculations were performed at high (~100 conidia per mm^2^) and low (~1 conidium per mm^2^) densities. Inoculation densities were assessed by placing 1 cm^2 ^cover slips, coated with agar, among plants to be inoculated and then counting the number of conidia per mm^2 ^by light microscopy. Unless stated otherwise, macroscopic disease development and lesion formation was monitored at 7 dpi. The extent of *G. cichoracearum *growth was quantified by one of two methods. At low inoculation densities, the total hyphal length of individual fungal colonies was measured periodically up to 4 dpi and the total number of conidiophores per colony were counted at 5 dpi [37]. At high inoculation densities, the number of mature conidiophores per field of view (0.16 mm^2^) was determined at 7 dpi. In each case, pictures of five randomly chosen fields of view per leaf and a minimum of 10 leaves per experiment were used to assess fungal growth. Growth of *H. parasitica *was monitored as described in [37]. All experiments were conducted at least twice.

### Staining, imaging and microscopy

Using 3-week old plants at 7 dpi, areas of healthy, chlorotic (yellow) and dead tissues were measured on 15 leaves of each treatment from photographs taken with a Spot Camera (Diagnostic Instruments, Inc.) attached to a dissecting microscope (Leica Wild M8, Leica Instruments, Inc., Exton, PA, USA). The photographs were imported into Photoshop 5.0 (Adobe, San Jose, CA, U.S.A.) and the "magic wand tool" was used to decompose the image into three components: green tissue (healthy tissue); yellow tissue (chlorotic tissue) and brown tissue (lesions and necrotic tissue). The area of each of these three components was measured with NIH IMAGE software 1.6267 [43] and used to calculate the percentage of total leaf area corresponding to each of the three components.

The staining method used to visualize fungal colonies and the software program employed to measure total hyphal length were described previously [37]. Microscopic lesions and fungal structures were visualized by staining the leaves with trypan blue [37]. Callose staining with aniline blue and the visualization of autofluorescent compounds were described in Adam and Somerville [44]. Hydrogen peroxide was visualized with 3,3'-diamino benzidine-HCl [45].

T3 transformants of *edr2-6 *containing the pCH2 construct encoding the *EDR:HA:GFP *driven by the *EDR2 *promoter were germinated on nutrient agar plants containing Musashige and Skoog salts 4.3 g per L, pH 5.7,1.5% agar, and hygromycin (30 μg per mL) (Sigma, St. Louis, MO). At 1 week after germination, plants expressing GFP were observed under a Leica DMIRE2 spinning disk confocal laser scanning microscope (Leica Microsystems, Inc.). The seedlings were mounted in water and excited with an Argon laser (488 nm) for eGFP visualization. Rosette leaves from 7-week-old plants were also observed. In addition, rosette leaves of 7-week-old EDR2:HA:eGFP expressing plants were stained with 4 μM MitoTracker Red CMXRos (Molecular Probes, Eugene, OR, USA) for 90 min. Collected image data sets were subsequently analyzed with the digital image analysis programs Image J (v. 1.30, N.I.H., USA) and Adobe Photoshop (v. 7.0).

### Abiotic treatments

Mechanical stresses were inflicted by wounding (e.g., cutting, puncturing, infiltrating with water or folding) mature leaves. Temperature stresses were performed at 4°C for 8 weeks or at 37°C for 6 or 15 h. The influence of the length of the day was tested by growing plants either in continuous light, or in conditions where the photoperiod was 14 or 10 h. The consequences of hydric stresses (drought, water saturated atmosphere for 24 h) were also recorded. Trypan blue staining of dead cells was performed at 1, 3 and 7 days after each treatment and visualized by light microscopy as described above. All tests were performed on 3-week-old plants and were repeated at least three times.

### Double mutant analysis

Standard genetic crosses were used to make double mutant lines of *edr2-6 *with *pad4-1 *[21], *ein2-1 *[46], *coi1-1 *[47] and the transgene *NahG *[48]. F2 plants homozygous for *edr2-6 *and *pad4-1 *or *edr2-6 *and *NahG *were identified by PCR [49]. The *ein2-1 *mutation was identified in plants by their lack of root growth inhibition when grown on Murashige and Skoog medium supplemented with 10 μM 1-aminocyclopropane-1-carboxylic acid. The *coi1-1 *mutation was identified in plants that did not exhibit a stunted growth habit when grown in Murashige and Skoog medium containing 20 μM methyl-jasmonate [50].

### Nucleic acid analysis and manipulations

The *edr2-6 *mutation was generated by inserting a 5.8 kb T-DNA fragment containing a right border (RB) and a left border (LB), the *BAR *gene, the *NPTII *gene and a fragment of an leucine-rich repeat gene driven by the 35S promoter. Given both that the phenotype of *edr2-6 *resembles that of the *edr2-1 *(a point mutation leading to the change, W256STOP) [16] and that the rescue experiment with the cloned *EDR2 *gene restored the wild-type phenotype to the *edr2-6 *mutant, we feel that the additional sequences in this construct did not contribute to the phenotypes described in the text. To test cosegregation of the *edr2-6 *mutation with the T-DNA, *edr2-6 *was crossed to Col-0 and 250 F_2 _plants were first analyzed for disease phenotypes 7 dpi with *G. cichoracearum *[37], and then scored for resistance to BASTA (25 μL glufonsinate ammonium per L) (Bayer Crop Sciences).

To clone *EDR2*, genomic regions flanking the T-DNA insert were amplified by PCR using the Universal Genome Walker Kit (Clontech, Mountain View, CA). The T-DNA insert-specific primers used were: LB 5'-AAC TTG ATT TGG GTG ATG GTT CAC GTA GTG-3', LB nested 5'-GCC CTG ATA GAC GGT TTT TCG CCC TTT GAC-3', RB 5'-CAA TCC ATC TTG TTC AAT CAT GCG AAA CGA-3' and RB nested 5'-CGA CTT TTG AAC GCG CAA TAA TGG TTT CTG-3'. Two BAC clones (F13C5 and F16M6) encompassing the region of interest were used to subclone a wild-type copy of the gene that was disrupted by the T-DNA insert in the *edr2-6 *mutant. A 12 kb *Nco*I fragment encompassing the gene was cloned into the pCAMBIA1380 [51]. The construct was introduced into *Agrobacterium tumefaciens *and subsequently into *edr2-6 *plants [52]. Transgenic plants were tested in the T1 and T2 generations for powdery mildew resistance.

A fragment of *EDR2 *cDNA was amplified via RT-PCR, cloned, and sequenced. The primers used were complementary to the poly-A tail (5'-GGC CAC GCG TCG ACT AGT ACT TTT TTT TTT TTT TTT T-3') and a region about 50 nucleotides before the predicted *EDR2 *start codon (5'-CCG TGG GGA AGT TTT GTG-3').

An EDR2:HA:GFP fusion construct under the control of the *EDR2 *native promoter was created. A fragment containing 1.4 kb upstream of the predicted translational start of *EDR2 *and 2.1 kb *EDR2*:HA cDNA were PCR amplified or RT-PCR amplified, respectively and fused together via two-template PCR using primers 5'-GCA GTC GAC GGT ACC AAT TCT GAC AGG TGC AGC TTT TCC-3' and 5'-CGG TCG AGA CCC GGG GAG CAT AAT CTG GAA CAT CGT ATG GAT AGC CTC CTG ACT CCA GAT TCG GAA C-3' [53]. The two templates were generated with primers 5'-GCA GTC GAC GGT ACC AAT TCT GAC AGG TGC AGC TTT TCC -3' and 5'-GAT CTT CCT CCT TCC ATA CCT AA-3' (promoter) and 5'-AAA TCT TCG CTA ATC GCA GAG AC-3' and 5'-CGG TCG AGA CCC GGG GAG CAT AAT CTG GAA CAT CGT ATG GAT AGC CTC CTG ACT CCA GAT TCG GAA C-3' (*EDR2 *cDNA with HA tag). The promoter (EDR2):EDR2 cDNA:HA construct was subsequently cloned into the *Kpn*I/*Sma*I sites of pSK001H to obtain pCH2. The plasmid pSK001H, which contained the gene for eGFP, was derived from pEZR(H)-NL by removing a *Sac*I fragment containing the CaMV 35S promoter. Plasmid pZEZR(H)-NL was provided by Dave Ehrhardt (Carnegie Institution, Stanford, CA) [54]. The plasmid pCH2 was introduced into *edr2-6 *mutants via Agrobacterium-mediated transformation [52] and T1 transformants were selected on hygromycin plates.

RNA extraction and northern blot analysis, using 15 μg of total RNA, were performed as described [55]. Signals were detected using a PhosphorImager (Typhoon 8600) and quantified using the ImagQuant program (Molecular Dynamics, Sunnyvale, CA). Additional information about the expression of *EDR2 *and related genes was recovered from Genevestigator [19,56].

Information about the EDR2 protein structure was recovered from TAIR, the Arabidopsis Information Resource [57], and from SUBA, the Subcellular Location of Proteins in Arabidopsis database [18,58]. Domains present in EDR2 were identified with the Hidden Markov Model program [59]. Proteins from other organisms that contained the same domains as EDR2 (PH, START, DUF1336 or PH, START) were identified using the Conserved Domain search provided by NCBI [28,60].

### Phosphoinositide binding assays

A fragment encoding the first 191 amino acids of EDR2 was amplified via PCR using the primers 5'-GCG GGA TCC ATG TCT AAG GTA GTG TAC GAA-3' and 5'-CCG GAA TTC TGG TTC GCC AAC TCT GCA TCA A-3'. This fragment was cloned into the *Bam*HI and *Eco*RI sites of the vector pGEX-2TK (Amersham Biosciences; Piscataway, NJ) and transformed into the *E. coli *strain BL21-CodonPlus(DE3)-RP (Stratagene; La Jolla, CA). Protein expression was induced with 1 mM isopropyl-β-D-thiogalactoside for 3 h. The purification of the glutathione-S-transferase (GST)-fusion protein was done according to the method described by Smith and Johnson [61] except that 1 M urea was included in the elution buffer.

PIP strips from Echelon Biosciences Inc. (Salt Lake City, UT) were blocked in TBS+M (10 mM Tris, HCl pH 7.0, 150 mM NaCl, 5% (w/v) milk powder) for 1 h and incubated with 0.05 mg/ml of the GST fusion protein in TBS+M for 1.5 h. The membranes were washed 4 times for 5 min with TBS + 0.05% (v/v) Tween20 and 2 times for 5 min with TBS. Incubation with the anti-GST (diluted 1:1000) and the anti-mouse antibody (1:7500) (both from Sigma, St. Louis, MO) were made for 1 h each in TBS+M with washing steps after each incubation step as described. Signals were detected with the SuperSignal West Pico Chemiluminescent Substrate from Pierce (Rockford, IL).

## Abbreviations

BTH: benzothiadiazole, dpi: days post-inoculation,eGFP, enhanced green fluorescent protein,GFP: green fluorescent protein, GST: glutathione-S-transferase, hpi: hours post-inoculation, HR: hypersensitive response, PH: pleckstrin homology domain, PI-4-P: phosphatidylinositol-4-phosphate, SA: salicylic acid, START: StAR (steriodogenic acute regulatory protein)-related lipid transfer domain

## Authors' contributions

JV performed the mutant screen and isolated the *edr2-6 *mutant. C. Schiff cloned the *EDR2 *gene and performed the characterization of the *edr2-6 *mutant, including the data presented in Figures 2, 3, 4, 5. MS performed the double mutant analysis and some of the infection studies presented in Figures 1, 2. SK and MN generated the EDR2-GFP fusion constructs and SK performed the confocal imaging presented in Figure 8. SV constructed the PH-GST fusion protein and determined the binding preference of the PH domain as illustrated in Figure 7 and performed some of the double mutant analysis presented Figures 1, 2. C. Schiff, MS, SV, C. Somerville and SS each contributed drafts of the manuscript and JV, SK and MN provided helpful comments on the manuscript.
